# Sulbactam-durlobactam: A Step Forward in Treating Carbapenem-Resistant *Acinetobacter baumannii* (CRAB) Infections

**DOI:** 10.1093/cid/ciad093

**Published:** 2023-05-01

**Authors:** Richard R Watkins, Robert A Bonomo

**Affiliations:** Department of Medicine, Division of Infectious Diseases, Northeast Ohio Medical University, Rootstown, OH, USA; Research Service, VA Northeast Ohio Healthcare System, Cleveland, OH, USA; Case Western Reserve University-Cleveland VA Medical Center for Antimicrobial Resistance and Epidemiology (Case VA CARES), Cleveland, OH, USA

**Keywords:** antimicrobial resistance, carbapenem-resistant *Acinetobacter baumannii*, sulbactam-durlobactam

## Abstract

Antimicrobial resistance in gram-negative pathogens, such as *Acinetobacter baumannii*, is a serious threat to human health. Sulbactam-durlobactam, a unique β-lactam and a β-lactamase inhibitor combination, is a novel agent targeted against carbapenem-resistant *A. baumannii*. This supplement provides a summary of the development of SUL-DUR, discussing its unique features and role in treating infections caused by CRAB pathogens.

The ongoing spread of antimicrobial resistance is an existential threat to modern medicine and humankind itself. In 2019, approximately 4.95 million deaths worldwide were associated with bacterial antimicrobial resistance [1]. Of all the bacterial pathogens responsible for human infections, carbapenem-resistant *Acinetobacter baumannii* (CRAB) is one of the most concerning. The Centers for Disease Control and Prevention has labeled CRAB an “urgent threat,” the highest level category [2]. The increased mortality from CRAB infections and the limited treatment options that are currently available contribute to this concern [3]. Indeed, most CRAB isolates are either extremely drug resistant (susceptible to only polymyxins, aminoglycosides, or tigecycline) or multidrug resistant, making treatment extremely challenging [4]. Patients infected with CRAB have at least twice the risk of dying compared with patients with carbapenem-susceptible *A. baumannii* strains [5]. Most CRAB infections occur in hospitalized patients, particularly those admitted to intensive care units and who often have multiple medical comorbidities. However, a number of infections do occur in the community (Asia Pacific) and long-term acute care settings [6, 7].

Carbapenem resistance in *A. baumannii* is primarily caused by the horizontal acquisition of class D (OXA-type) carbapenemase genes [8]. Other mechanisms of carbapenem resistance include the acquisition of class B (VIM-, IMP-, and NDM-type) carbapenemases, loss of the outer membrane protein CarO, and modifications of the AdeABC resistance nodulation division efflux pump [9]. These latter mechanisms of resistance are relatively uncommon and do not contribute to this menacing phenotype to the extent as do the class D carbapenemase genes.

Colistin, a cationic antimicrobial peptide antibiotic of the polymyxin class, fell out of clinical use in the 1970s because of a high incidence of adverse events and the availability of safer and equally potent carbapenems and cephalosporins. Unfortunately, polymyxins were desperately resurrected as “salvage” or “last resort” therapy with the appearance of multidrug resistant gram-negative bacteria, including CRAB, in the early years of the 21st century [10]. As anticipated, resistance to colistin quickly emerged. More recently, cefiderocol was approved for the treatment of hospital-acquired bacterial pneumonia and ventilator-associated bacterial pneumonia caused by gram-negative organisms, including *A. baumannii* [11]. Despite apparent stability to hydrolysis against the chromosomal *Acinetobacter*-derived cephalosporinase, the development of cefiderocol-resistant *A. baumannii* strains resulting from mutations in iron transport complex, β-lactamases, or penicillin-binding proteins (PBPs) has already been reported [12]. Moreover, the treatment outcomes of patients with CRAB infection treated with cefiderocol was disappointing, with a mortality imbalance in favor of the comparator (best available therapy) [13]. Despite potent in vitro antimicrobial activity against CRAB, tigecycline has a number of disadvantages including poor distribution in tissue, low plasma concentration, and adverse reactions, particularly of the gastrointestinal tract. Therefore, new treatment options for CRAB are urgently needed.

Sulbactam was initially developed by Pfizer as a sulfone β-lactamase inhibitor [14]. Sulbactam was also found to be a cell wall synthesis inhibitor that blocks essential PBPs in *Acinetobacter* spp. and a small number of other Gram-negatives (*Neisseria* spp.), leading to cell death. Usually coformulated with ampicillin, sulbactam is commonly used in combination with other antibiotics against CRAB. These include: (1) polymyxin-B-meropenem-sulbactam; (2) polymyxin-B-meropenem-ampicillin-sulbactam; (3) colistin-doripenem-sulbactam; (4) colistin-sulbactam; (5) polymyxin-B-sulbactam; (6) polymyxin-B-sulbactam-meropenem; (7) polymyxin-B-ampicillin-sulbactam-meropenem; and (8) and colistin-sulbactam-doripenem [15]. Wang et al. demonstrated that the higher the ratio of sulbactam, the more potent in vitro antimicrobial activity of imipenem-sulbactam, ampicillin-sulbactam, and cefoperazone-sulbactam combinations against *A. baumannii* [16]. Furthermore, cefoperazone-sulbactam at a combination of 1:3 showed the highest in vitro activity among all sulbactam-based combinations and had superior activity to most comparator agents.

The mechanisms by which sulbactam and other β-lactam combinations act against CRAB is not entirely well-studied but may be due to a favorable 50% inhibitory concentration of sulbactam for PBP3 [17]. Furthermore, when other β-lactams are coadministered, the role of inhibiting multiple PBPs simultaneously has not yet been fully appreciated. Sulbactam itself is subject to degradation by a wide variety of β-lactamases found in *Acinetobacter* spp. [18]. Thus, combining sulbactam with a novel, broad-spectrum β-lactamase inhibitor is a mechanistically sound approach to deal with the ongoing scourge of CRAB.

Durlobactam (DUR) (ETX2514) is a novel broad-spectrum diazabicyclooctane (DBO) non–β-lactam β-lactamase inhibitor. A number of studies have established that DUR is a highly potent DBO β-lactamase inhibitor, with an expanded spectrum compared with other DBO inhibitors, which also possesses intrinsic antibacterial activity on its own against some bacterial species [19–21]. Interestingly, DUR binds to PBPs, as evident in studies demonstrating rapid acylation rate constants and phase contrast microscopy studies in which there is a major morphological effect (formation of spherical forms suggesting PBP2 inhibition), although this impact on *Acinetobacter* minimum inhibitory concentrations (MICs) is not evident when tested alone [19]. To its specific role against CRAB, DUR is a highly potent inhibitor of *Acinetobacter*-derived cephalosporinase and class D β-lactamases including carbapenemases of the OXA family (OXA-23, OXA-24/40), which are prevalent in CRAB.

The basic elements of a potentially effective therapy to address CRAB are present in the combination of sulbactam with durlobactam (sulbactam-durlobactam; SUL-DUR). A phase 2 randomized clinical trial enrolled patients with complicated urinary tract infections (albeit none resulting from *A. baumannii*) and randomized them 2:1 to receive SUL-DUR or placebo for 7 days along with background therapy with imipenem-cilastin to evaluate the tolerability of SUL-DUR in hospitalized patients [22]. SUL-DUR with imipenem-cilastin was well tolerated with no serious adverse events reported, and the pharmacokinetics of SUL-DUR was similar to that observed in healthy volunteers. A recently completed phase 3 trial (ATTACK) compared SUL-DUR with colistin for treating serious infections resulting from carbapenem-resistant *A. baumannii*–*calcoaceticus* complex (ABC) [23]. SUL-DUR met the primary noninferiority endpoint of 28-day all-cause mortality. In the ATTACK trial, SUL-DUR had a favorable safety profile, with a statistically significant lower incidence of nephrotoxicity compared with colistin.

Although clinical trial experience with SUL-DUR is limited, early case reports appear promising. Zaidan et al. reported a case of a patient with extensively-drug resistant *A. baumannii* pneumonia and septic shock who was successfully treated with a combination of cefiderocol and SUL-DUR obtained through an emergency investigational new drug application [24]. However, because of the concomitant dosing of cefiderocol, it is not possible to know which agent was the primary driver of microbial eradication. The authors hypothesized that DUR, in addition to providing protection for SUL, may have also enhanced the activity of cefiderocol. The mechanistic basis for this success may also parallel the ATTACK trial, in which combinations and hitting multiple targets enhanced efficacy [23].

This supplement to *Clinical Infectious Diseases* highlights the unique properties of SUL-DUR and discusses its potential role in treating infections resulting from CRAB. The first article by Castanheira and Gales describes the epidemiology and mechanisms of resistance in ABC organisms, including CRAB. This is a timely and important topic given that a recent study from 31 countries across Asia/South Pacific, Europe, Latin America, the Middle East, and North America found more than 50% of ABC isolates were resistant to carbapenems [25]. In the second article, Shields et al. detail the current treatment options available, along with unmet medical needs. Next, the article by Papp-Wallace et al. discusses the structural studies on DUR and provides an up-to-date understanding of how the chemical structure of the drug impacts its functionality. The discovery of DUR is described, as are the in vivo and in vitro activity of SUL-DUR and mechanisms of resistance to SUL-DUR. The molecular basis for the efficacy of the combination against *A. baumannii*, with an emphasis on the biochemical characteristics of each partner, is further discussed. The pharmacokinetics and pharmacodynamics of SUL-DUR is the topic of the following article by Bhavnani and O’Donnell. In the final article, Watkins et al. discuss the recently completed ATTACK trial that compared SUL-DUR with colistin for treating serious infections resulting from carbapenem-resistant ABC.

Resistance to antibiotics is an inevitable and natural consequence of bacterial evolution that follows exposure to these agents. Identifying and developing therapeutics that can disrupt this biological process is of paramount importance. Thus, SUL-DUR, if approved, represents a very welcome addition to our antibiotic armamentarium. Its novel characteristics and encouraging clinical data suggest SUL-DUR will likely play a role in the management of infections caused by CRAB. From a mechanistic perspective, further investigations are needed to elucidate its place in therapy and to further define whether other β-lactams, by inhibiting multiple PBPs like imipenem and cefiderocol, add to its efficacy.

## Notes

***Financial support*.** This work was supported by Entasis Therapeutics, a wholly owned subsidiary of Innoviva, Inc. The authors did not receive any fees for authorship.

***Supplement sponsorship*.** This article appears as part of the supplement “Sulbactam-durlobactam, a Targeted β-lactam/β-lactamase Inhibitor, for MDR *Acinetobacter*,” sponsored by Entasis Therapeutics Inc., a wholly owned subsidiary of Innoviva, Inc.
